# Effects of Cardiac Telerehabilitation During COVID-19 on Cardiorespiratory Capacities in Patients With Coronary Artery Disease

**DOI:** 10.3389/fphys.2022.837482

**Published:** 2022-03-17

**Authors:** Marie Fanget, Manon Bayle, Pierre Labeix, Frédéric Roche, David Hupin

**Affiliations:** ^1^INSERM, U1059, SAINBIOSE, DVH, University of Lyon, Jean Monnet University, Saint-Etienne, France; ^2^Department of Clinical and Exercise Physiology, University Hospital Center of Saint-Etienne, Saint-Etienne, France; ^3^Department of Medicine, K2, Solna, Karolinska Institutet, Stockholm, Sweden

**Keywords:** telerehabilitation, physical activity, coronary artery disease, COVID-19, cardiorespiratory fitness, exercise training, cardiac rehabilitation

## Abstract

**Background:**

The COVID-19 pandemic led to the closure of most cardiac therapy centers. One of the solutions was to adapt the existing cardiac rehabilitation (CR) program in an institute to a remote approach offered by home-based telerehabilitation. The aim of this study was to measure the cardiorespiratory effects of telerehabilitation compared to conventional center-based CR.

**Methods:**

Patients were assigned to two 3-week CR programs: telerehabilitation and conventional center-based CR. The telerehabilitation group wore a connected watch to monitor heart rate (HR) and gave their perception of effort according to a modified Borg scale. The exercise training (four sessions/week) consisted of 1-h aerobic endurance and strength training session at the target HR zone determined by results based on cardiopulmonary exercise test (CPET) and perception of effort, respectively. The exercise protocol was the same for conventional CR participants except the duration of session that lasted 2 h instead of one. The week before and after the training program, peak oxygen uptake (VO_2_ peak), oxygen uptake at first ventilatory threshold (VO_2_ at VT_1_), peak workload, percent of predicted maximum HR, and the absolute differences in HR and systolic blood pressure between maximum and recovery at 1 and 3 min were measured using a CPET. A two-way ANOVA with one repeated measure and one independent factor was performed.

**Results:**

Fifty-four patients (mean age: 61.5 ± 8.6 years, 10 women) equally split in the two groups were included in this experiment. A significant increase was observed in both groups on VO_2_ peak (telerehabilitation: 8.1 ± 7.8% vs. conventional: 10.1 ± 9.7%, *p* < 0.001), VO_2_ at VT_1_ (telerehabilitation: 8.8 ± 4.4% vs. conventional: 7.3 ± 19.0%, *p* = 0.02) and peak workload (telerehabilitation: 16.6 ± 18.9% vs. conventional: 17.2 ± 7.0%, *p* < 0.001) after the 3-week telerehabilitation and conventional CR, respectively. No significant difference was noticed between both groups.

**Conclusion:**

A 3-week exercise program improved patients’ cardiorespiratory fitness. Telerehabilitation was as effective and represents a safe alternative CR program during the COVID-19 period. In the future, this approach could facilitate the continuity of care for patients unable to participate in center-based CR.

## Introduction

Coronary artery disease, one of the most common cardiovascular (CV) diseases, accounts for a large proportion of deaths worldwide (Roth et al., 2017). Secondary prevention consists of decreasing as much as possible all CV risk factors in order to avoid the recurrence of cardiac events (Ades, 2001). Although preventive drug therapy is a priority after myocardial infarction, patients suffer from neuromuscular deconditioning, dyspnea, and poor quality of life (Cavalheiro et al., 2021). To restore or increase physical abilities and reduce CV risk, a cardiac rehabilitation (CR) program is required after myocardial infarction (Iliou et al., 2015). A predominant part of CR is physical exercise (Balady et al., 2007; Price et al., 2016; Ambrosetti et al., 2021). Nevertheless, a holistic management strategy is recommended (Balady et al., 2007). In addition to training, programs provide behavioral changes and lifestyle therapeutic education on coronary artery disease risk factor management and psychological assistance (Ambrosetti et al., 2021). The objective for active subjects is to regain their place in society and for older persons to maintain their independence (Pavy et al., 2012; Iliou et al., 2015). The benefits of CR are actually well described in the literature (Wisløff et al., 2007; Scrutinio et al., 2009; Piepoli et al., 2016). These include increased functional, muscular and cardiopulmonary capacities and also greater control of CV risk factors by adopting a better lifestyle, such as smoking cessation, a heart-healthy diet, and stress management (Scrutinio et al., 2009).

In December 2019, severe acute respiratory syndrome coronavirus-2 (SARS-CoV-2) was discovered in China (Pericàs et al., 2020). Three months later, the World Health Organization declared the SARS-CoV-2 disease (COVID-19) a pandemic (Cucinotta and Vanelli, 2020). This virus spread very rapidly throughout the world and caused many economic, social, and health consequences (Pericàs et al., 2020). The latest repercussion led to the saturation of hospital services which were forced to close rehabilitation centers (Taylor et al., 2021). Therefore, in many institutes or specialized CR clinics, the programs were partially interrupted or suspended according recommendations of scientific and public health authorities (Haute Autorité de Santé, 2020; Ministère des Solidarités et de la Santé, 2020). An alternative CR delivery strategies should be used to remedy these barriers.

One of the solutions was to adapt the existing center-based CR program to a remote approach offered by telerehabilitation (Chan et al., 2016). Telerehabilitation is medical technology-assisted delivery model to provide healthcare services between healthcare professionals and home-based patients (Silva-Cardoso et al., 2021). This therapy includes remotely supervised exercise training and collective or individual cardiac prevention and management heart disease meetings by videoconference (Scherrenberg et al., 2021). To demonstrate the effectiveness of cardiac telerehabilitation, some studies have focused on quality of life, mainly assessed by questionnaires (Gooley et al., 2021; Taylor et al., 2021). Patients also suffer from physical limitations such as shortness of breath, lack of fitness, and fatigue during exercise. Hence, one of the priorities is to improve physical capacity.

The aim of this study was to investigate and measure the effects of home-based CR compared to conventional center-based CR on cardiorespiratory functions in coronary artery disease patients. We hypothesized that telerehabilitation would be as effective as traditional CR realized in a conventional hospital setting.

## Materials and Methods

### Inclusion Criteria

Firstly, we controlled the low risk of patients experiencing an adverse event during CR. We based this on their postsurgical or medical intervention complications (no complications), asymptomatic, no ventricular arrhythmias, no heart failure, no left ventricular dysfunction, and test results as CV response during the cardiopulmonary exercise test (CPET) before the CR.

All participants had to be over 18 years old, had acute coronary syndrome treated within the last 6 months, had received coronary revascularization by percutaneous coronary intervention (angioplasty with stent implantation) or surgical operation (coronary artery bypass grafting). The exclusion criteria were uncontrolled ventricular rhythm disorders and articular or respiratory diseases.

In addition to meeting the above inclusion criteria, patients who followed the home-based CR program were requested to have internet access and an indoor exercise bike at home but were excluded if they had significant deconditioning that required on-site supervision.

All volunteers provided written informed consent before beginning the experimentation. The study was in accordance with the Declaration of Helsinki and the protocol was approved by the Ethics Committee of the university hospital of Saint-Etienne, France (IRBN1022021/CHUSTE).

### Experimental Design

**FIGURE 1:**
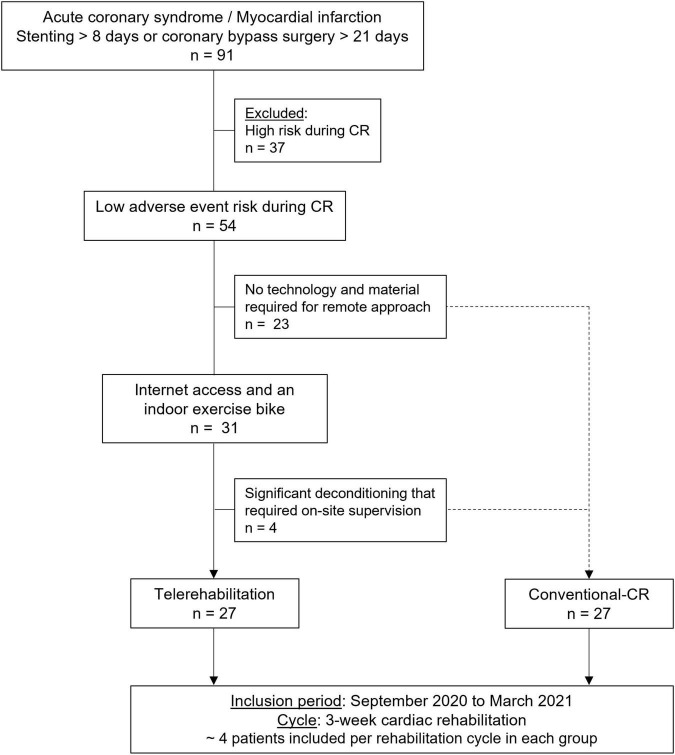
Decision flow chart. CR, cardiac rehabilitation.

The non-randomized investigation was conducted from September 2020 to March 2021 in a single rehabilitation center (Saint-Etienne University Hospital, Saint-Etienne, France). CR was offered as soon as possible after 8 d of coronary intervention (Corbett et al., 2021). Eligible patients were assigned to two groups: home-based CR (telerehabilitation) and traditional center-based CR (conventional CR). Each group was composed of 4 patients per CR cycle (Figure 1). This multidisciplinary medical and paramedical care was offered to patients for 3 weeks. Exercise training represented an essential part of this CR since participants practiced four consecutive sessions of physical activity per week. The last day of the week was devoted to a group therapeutic education workshop. Before starting the intervention, patients were interviewed by a specialist nurse about their CV risk factors and personal objectives. According to their needs and in addition to exercise program and collective meetings, a medical, diet, psychology and/or tobacco expert could be individually proposed by videoconference or face-to-face according to the method of rehabilitation (McDonagh et al., 2021). At the end of CR, recommendations and guidance were given by physical activity specialist to maintain exercise training independently.

### Physiological Assessments

Elementary anthropometric measurements such as body mass and body mass index (BMI) were assessed before and after the 3-week CR. BMI was calculated by dividing body mass (in kg) by the square of body height (in m).

An initial transthoracic echocardiography was carried out to define the left ventricular ejection fraction. Patients repeated this test only if an abnormality was detected the first time.

The week before and after the intervention program, the patients performed in hospital a maximum CPET on an electronically braked ergocycle (Vyntus CPX, CareFusion, San Diego, CA, United States). Two experienced medical doctors specialists in exercise physiology (DH, FR) used a ramp-type protocol, consisting of 2 min warming up to 10 W, followed by a 10-W progressive increment every minute until exhaustion (Writing Committee et al., 2012). The automated metabolic system analyzed respiratory gas exchange including oxygen uptake (VO_2_). The average temperature and relative humidity in testing room were 21°C and 24%, respectively. Peak oxygen uptake (VO_2_ peak) was determined as the mean value of the last 30 s of exercise. VO_2_ at the first ventilatory threshold (VO_2_ at VT_1_) and the peak workload (PWL) were evaluated to illustrate the achievable efforts without dyspnea such as carrying out tasks of daily life without difficulty and the duration of the exercise, respectively (Writing Committee et al., 2012).

Participants were monitored continuously with electrocardiography (ECG). Hence, the heart rate (HR) was recorded. Systolic blood pressure (SBP) was measured manually by an experienced nurse using a random-zero sphygmomanometer when the participant was sitting on the cycle ergometer every 2 min during exercise, and at 1 and 3 min recovery from exercise.

More specifically, we focused on the percentage of the predicted maximum HR (%HRpeak = the ratio of peak measured HR and peak predicted HR). HR recovery (HRR) was measured at 1 and 3 min following peak HR during exercise. Peak HR was identified as the maximum HR during the exercise protocol. HRR 1 min (ΔHRR 1 min) was defined as the absolute change from peak HR to HR 1 min post peak HR (HRR1 = peak HR – HR at 1 min post peak HR) (Shetler et al., 2001; Arduini et al., 2011). Similarly, HRs of recovery 3 min (ΔHRR 3 min) was calculated as the absolute change from peak HR to HRs 3 min post peak HR (Peçanha et al., 2017). Maximal SBP was the highest value achieved during the exercise ECG. SBP recovery deltas between maximal and at 1-min (ΔSBP 1 min) and 3-min (ΔSBP 3 min) recovery from exercise were also measured.

### Cardiac Telerehabilitation

**FIGURE 2:**
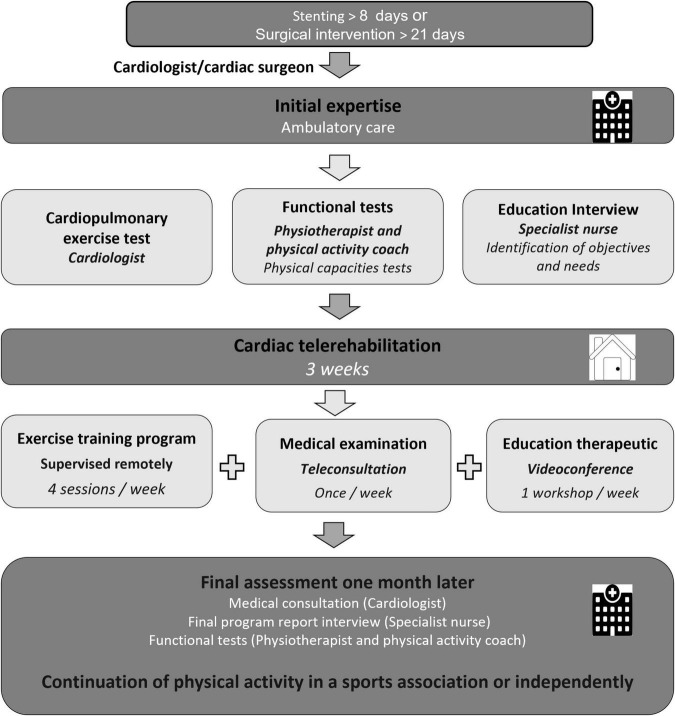
Experimental design of telerehabilitation intervention.

The telerehabilitation process is described in Figure 2. Medical and paramedical teleconsultations were carried out by videoconference. Before the start of exercise training, the personal coach checked the correct functioning of network connection and presented the exercise training program. Patients performed 1-h physical activity sessions at home using digital technology available to them (computer or tablet). The training session consisted of 30 min of cycling and 20 min of strength training. The physical exercise program, the frequency and the intensity were the same for the conventional CR group, except the duration of training session lasted twice as long. To ensure safety and adapt the intervention for each participant, they wore a connected watch (Dona Care, Life Plus, Versailles, France) to monitor the HR and assess the number of steps per day during the program. They gave their perceived exertion using a modified Borg scale from 0 (no sweating, no shortness of breath, no exertion at all) to 10 (extremely hard exertion). In addition, before each training session, the adapted physical activity coach asked the patients about their fatigue or if they had pains.

### Therapeutic Education Meetings

Once a week, a therapeutic education meeting was organized. These educational and recreational thematic workshops allowed to deal with the difficulties linked to a patient’s pathology and to discuss the main issues related to their cardiac event (Janssen et al., 2013; Pavy et al., 2013). Thanks to these meetings, participants developed their knowledge of their CV disease and the options to reduce their CV risk factors. A first workshop was led by a cardiologist on cardiac medical intervention and pharmacological treatment. A dietician coordinated the second seminar relative to heart-healthy nutrition. The last topic by a physiotherapist was on CPET and physical activity.

### Physical Exercise Program

**FIGURE 3:**
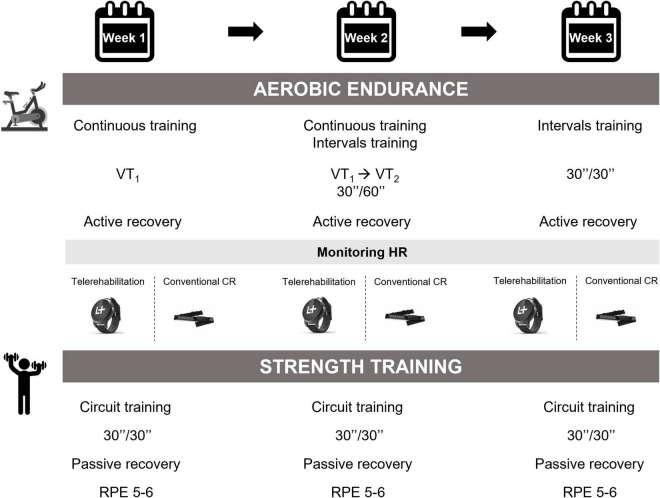
Physical exercise intervention. VT, ventilatory threshold; HR, heart rate; CR, cardiac rehabilitation; RPE, rating of perceived exertion.

Figure 3 illustrates the physical training program. The first half of the training session on a cycle ergometer was devoted to aerobic endurance. The intensity of this prolonged submaximal exercise was constant and adapted accordingly to the CPET results of each patient. More specifically, the HR at VT_1_ defined the intensity of endurance training session (Ambrosetti et al., 2021). The physical exercise session included a warm-up period, a CV training period, and a cool down phase. In addition, an aerobic interval training was gradually offered as an alternative to continuous endurance exercise. The dynamic resistance training consisted of overall muscle strength training or focused more on the lower or upper limbs.

### Statistical Analysis

Statistical analyses were performed using JASP (version 0.15). All data were reported as mean ± standard deviation (SD). We checked distribution of normality and the homogeneity of variances with the Shapiro-Wilk and Levene tests, respectively. The effect of the training program on cardiorespiratory parameters according to CR approach was evaluated using two-way repeated measures ANOVA, i.e., CR groups (telerehabilitation vs. conventional center-based) x time (pre-post). Where a significant interaction difference occurred, Tukey’s *post hoc* analyses were performed. For all statistical comparisons, the level of significance was set at *p* < 0.05.

## Results

Fifty-four patients were recruited in this study (mean age: 61.5 ± 8.6 years, 11% women). Half of the patients completed conventional CR in hospital (*n* = 27). The second half followed the telerehabilitation program (*n* = 27). CV risk factors, cardiac intervention, medical treatment and baseline characteristics of both groups are described in Table 1. No significant differences between telerehabilitation and conventional CR groups were observed at baseline. No adverse events were occurred during the CR period and all participants completed the exercise intervention.

**TABLE 1:** Baseline characteristics of the participants.

	Telerehabilitation (*n* = 27)	Conventional CR (*n* = 27)	*p*-value
Age (years)	63.3 ± 8.1	59.7 ± 9.1	0.13
Females	4 (15)	6 (22)	0.50
Distance from hospital (km)	41.2 ± 30.9	31.8 ± 22.4	0.36
Body mass (kg)	80.3 ± 17.3	72.1 ± 16.9	0.08
BMI (kg m^–2^)	27.0 ± 4.5	25.2 ± 4.6	0.16
LVEF (%)	54.9 ± 7.5	54.7 ± 9.5	0.53
LVEF < 50%	4 (15)	7 (26)	0.32
Beta blocker	23 (85)	25 (93)	0.40
Aspirin	25 (93)	27 (100)	0.16
Double APT	26 (96)	26 (96)	1.00
Statin	26 (96)	27 (100)	0.34
ACEI/ARB	23 (85)	27 (100)	0.33
High blood pressure	8 (30)	7 (26)	0.77
Dyslipidemia	8 (30)	8 (30)	1.00
Diabetes	2 (7)	0 (0)	0.16
**Sleep apnea**			
Total	6 (22)	9 (33)	0.37
Whose detect during CR	2 (33)	5 (56)	
**Smoking status**			0.14
Never	10 (37)	5 (19)	
Former	17 (63)	22 (81)	
Current	2 (7)	4 (15)	
**Coronary artery intervention**			
Medical	3 (11)	0 (0)	0.24
Angioplasty (stenting)	21 (78)	23 (85)	0.22
CABG	3 (11)	4 (15)	0.70

*Values are mean ± SD or n (%). CR, cardiac rehabilitation; BMI, body mass index; CABG, coronary artery bypass grafting; APT, Anti-platelet; ACEI/ARB, angiotensin-converting enzyme inhibitor/angiotensin receptor blocker; LVEF, left ventricular ejection fraction.*

**FIGURE 4:**
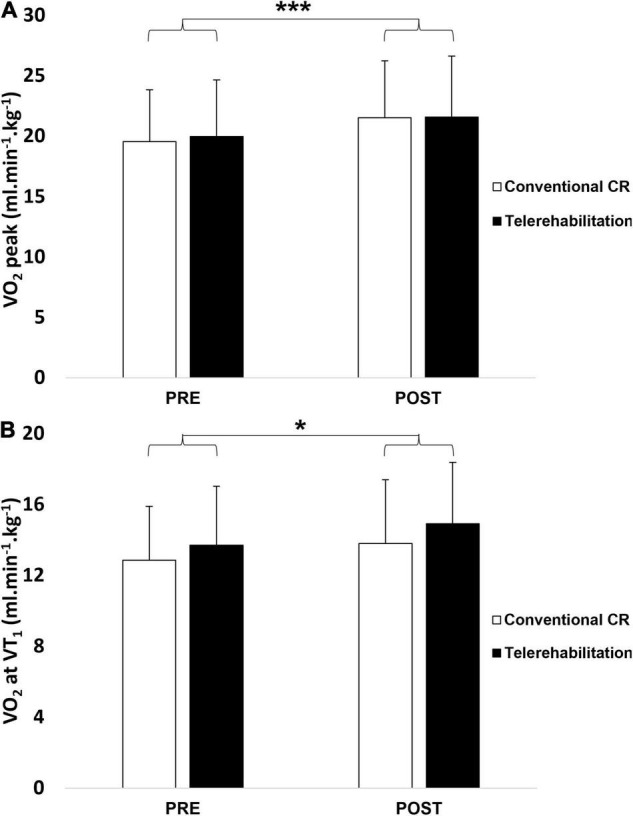
Peak oxygen uptake **(A)** and oxygen uptake at the first ventilatory threshold **(B)** before and after a 3-week exercise program between telerehabilitation (black bars) and conventional CR (white bars) groups. CR, cardiac rehabilitation; VO_2_ peak, peak oxygen uptake; VO_2_ at VT_1_, oxygen uptake at the first ventilatory threshold. *Significantly difference between baseline (*p* < 0.05); ***significantly difference between baseline (*p* < 0.001).

The results are presented in Table 2. Except for SBP parameters, we noticed a significant increase of all variables within both groups, while there was no significant difference between groups. VO_2_ peak and VO_2_ at VT_1_, are represented in Figure 4. The same conclusions were applicable for these physiological variables.

**TABLE 2:** Effect of 3-week exercise training on cardiorespiratory parameters between telerehabilitation and conventional cardiac rehabilitation program.

	Telerehabilitation (*n* = 27)		Conventional CR (*n* = 27)		Main time effect	Main group effect	Interaction effect
	Pre	Post	Pre	Post			
VO_2_ peak (L min^–1^)	1.6 ± 0.4	1.7 ± 0.5***	1.4 ± 0.4	1.5 ± 0.4***	< 0.001	0.124	0.984
VO_2_ peak (ml min^–1^ kg^–1^)	20.0 ± 4.7	21.6 ± 5.0***	19.5 ± 4.3	21.5 ± 4.7***	< 0.001	0.844	0.678
VO_2_ at VT_1_ (ml min^–1^ kg^–1^)	13.7 ± 3.3	14.9 ± 3.5*	12.9 ± 3.0	13.8 ± 3.6*	0.023	0.222	0.771
PWL (W)	123.9 ± 36.8	144.4 ± 43.7***	111.0 ± 34.1	128.1 ± 35.8***	< 0.001	0.156	0.419
HR peak (bpm)	123.7 ± 22.6	127.9 ± 23.0***	118.5 ± 18.9	130.9 ± 27.0***^†^	< 0.001	0.858	0.031
%HR peak (%)	79.7 ± 14.4	82.5 ± 14.8***	72.3 ± 18.3	82.8 ± 15.7***^†^	< 0.001	0.379	0.038
SBP max (mmHg)	176.3 ± 26.4	179.9 ± 29.3	169.8 ± 22.8	173.0 ± 24.5	0.266	0.304	0.947
ΔHRR 1 min (bpm)	17.9 ± 7.4	19.9 ± 8.2*	13.0 ± 12.2	18.0 ± 10.5*	0.035	0.129	0.361
ΔSBP 1 min (mmHg)	−1.5 ± 15.2	5.8 ± 22.0	4.7 ± 21.7	2.7 ± 22.1	0.530	0.714	0.277
ΔHRR 3 min (bpm)	34.0 ± 12.7	39.7 ± 13.9***	27.3 ± 9.4	40.8 ± 16.4***^†^	< 0.001	0.399	0.018
ΔSBP 3 min (mmHg)	18.6 ± 19.0	20.4 ± 23.7	15.2 ± 18.7	18.5 ± 22.1	0.518	0.846	0.552
Duration CPET (min)	9.1 ± 2.6	10.3 ± 2.5**	8.6 ± 3.0	9.5 ± 2.6**	0.007	0.300	0.691

*CR, cardiac rehabilitation; VO_2_ peak, peak oxygen uptake; VO_2_ at VT_1_, oxygen uptake at the first ventilatory threshold; PWL, peak workload; W, watts; HR, heart rate; HR peak, peak heart rate; %HR peak, ratio of peak measured HR out of peak predicted HR; bpm, beats per minute; SBP, systolic blood pressure; mmHg, millimeters of mercury; ΔHRR 1 min, difference between maximum heart rate and heart rate after 1 min of recovery; ΔHRR 3 min, difference between maximum heart rate and heart rate after 3 min of recovery; CPET, cardiopulmonary exercise test.*

*Significantly difference between baseline (p < 0.05).

**Significantly difference between baseline (p < 0.01).

***Significantly difference between baseline (p < 0.001);

*^†^Time × method of cardiac rehabilitation interaction.*

There were both time effect × CR method interaction for HR peak (*p* = 0.031), %HR peak (*p* = 0.038) and ΔHRR 3 min (*p* = 0.018) values. Tukey *post hoc* tests showed statistically significant time difference (pre vs. post) in conventional CR group for these two variables. In addition, we observed a significant difference between the conventional group at baseline and telerehabilitation group after 3-week training program only for ΔHRR 3 min parameter.

## Discussion

This study examined the cardiorespiratory benefits after a 3-week telerehabilitation compared to conventional CR in hospital. The main findings of this research were an improvement of VO_2_ peak, VO_2_ at VT_1_ and PWL, irrespective of the CR strategy.

Enhanced cardiorespiratory fitness is one of the main objectives after acute coronary syndrome (Price et al., 2016; Pelliccia et al., 2021). In our experimentation, patients who followed a telerehabilitation program increased VO_2_ peak, VO_2_ at VT_1_ and PWL, by 8.1, 8.8, and 16.6%, respectively. As expected, we achieved similar physiological improvements as the conventional center-based CR group since they increased by 10.1, 7.3, and 17.1%, respectively. The duration of the interventional CR strategy was shorter than other investigations (i.e., 3 weeks vs. 8–12 weeks) (Kraal et al., 2014; Batalik et al., 2020). This was due to a more intensive and frequent exercise training with four physical activity sessions per week. Therefore, the results were similar to several other studies (Karapolat et al., 2009; Kraal et al., 2014; Vysoký et al., 2015; Batalik et al., 2021).

Most studies which used telerehabilitation as an add-on to center-based CR or an alternative for conventional CR suggested that it was a safe and well tolerated approach for patients (Kraal et al., 2014; Taylor et al., 2015; Kikuchi et al., 2021). It was also the case in our study since all patients satisfactorily achieved this CR program and no adverse events were reported. Thanks to current technological advances (smartwatch, accelerometers, pedometers), it is easier to collect and record physiological constants in order to ensure the safety in home environment and adapt the physical training intervention (Frederix et al., 2015; Scherrenberg et al., 2021).

Moreover, one of the major issues of conventional center-based CR is the low adherence (Zhang et al., 2018). Telerehabilitation could increase the number of potential participations (Batalik et al., 2021). In France, despite reimbursed coverage and well organized post-infarction care, only 30% of patients benefit from a CR program. Indeed, remote technology assistance can overcome accessibility barriers such as socioeconomic and travel difficulties, professional constraints, geographical distance of CR center. The latter two obstacles represent the major limiting factors in France. Hence, telerehabilitation may be an additional feasible and effective solution due to better integration of CR into the daily life of patients.

In addition, Avila et al. (2020) assessed cardiorespiratory and strength variables after one-year follow-up and highlighted the preservation of exercise capacity for the telerehabilitation group.

The secondary outcome showed a time effect for all HR parameters as above cardiopulmonary values and an interaction (time × CR strategy) for the HR peak, %HR peak and ΔHRR 3 min. Traditional CR remains a reference and it appears to be more effective on the HR variables since a greater improvement of these findings was observed in conventional CR programs compared to telerehabilitation. The postexercise HR response recovery could serve to assess autonomic nervous system activity. The recovery HR reflects autonomic nervous system activity after the exercise phase and more specifically the reactivation of parasympathetic tone after cessation of effort (Peçanha et al., 2017). A slow decline in HR after exercise suggests non-optimal parasympathetic and orthosympathetic balance or cardiac autonomic impairment. It is also a strong independent marker of CV mortality (Lipinski et al., 2004; Jouven and Courbon, 2005). Physical exercise training in CR optimizes the recovery kinetics postexercise. This is associated with improved findings (Jolly et al., 2011).

Although some HR variables which reflect autonomic nervous system components indicated lower improvements with the telerehabilitation program and suggested to favor a center-based CR, the various scientific evidence show that remote technology services can be proposed to meet the objectives of CR.

### Strengths and Limitations

We emphasized that our investigation is one of the first studies conducted in France on the use of cardiac telerehabilitation during the COVID-19 pandemic with objective physiological measurements (few self-reported parameters). In addition, the patients followed a holistic cardiac telerehabilitation, i.e., lifestyle counseling, clinical examinations and exercise training.

We should add an autonomic nervous system measurement to illustrate an improvement in the parasympathetic part and a decrease in the orthosympathetic system. One limitation of the present research study was the non-measurement of telerehabilitation long-term effects. The duration of training session was not the same between the two groups. Telerehabilitation performed the exercise training program without interruption, while patients who followed a standard center-based CR had longer rest period. Furthermore, this time included the changing room, general health checking, physiological measurements like oxygen saturation and blood pressure. Our investigation was not a randomized study, was in a single rehabilitation hospital center and a control group was absent. To validate these findings, we need to propose a multicenter randomized controlled trial.

## Conclusion

Following a 3-week exercise intervention effectively improved cardiorespiratory capacities in coronary artery patients. This investigation showed that telerehabilitation might become a relevant alternative to conventional center-based CR. This innovative healthcare delivery method appears to be a feasible, tolerable, safe and cost-effective solution. In the future, this approach could facilitate the continuity of care for people who encounter geographical or social accessibility difficulties.

## Data Availability Statement

The original contributions presented in the study are included in the article/supplementary material, further inquiries can be directed to the corresponding author.

## Ethics Statement

The studies involving human participants were reviewed and approved by Comité d’Ethique du CHU de Saint-Etienne IRBN1022021/CHUSTE. The patients provided their written informed consent to participate in this study.

## Author Contributions

MF and DH contributed to the conception or design of the study. MF, FR, and DH contributed to the analysis and/or interpretation of data. MF drafted the manuscript. MB, PL, FR, and DH critically revised the manuscript. All authors gave final approval and agreed to be accountable for all aspects of work ensuring integrity and accuracy.

## Conflict of Interest

The authors declare that the research was conducted in the absence of any commercial or financial relationships that could be construed as a potential conflict of interest.

## Publisher’s Note

All claims expressed in this article are solely those of the authors and do not necessarily represent those of their affiliated organizations, or those of the publisher, the editors and the reviewers. Any product that may be evaluated in this article, or claim that may be made by its manufacturer, is not guaranteed or endorsed by the publisher.

## References

[B1] Ades P. A. (2001). Cardiac Rehabilitation and Secondary Prevention of Coronary Heart Disease. *N. Engl. J. Med.* 345:892–902. 11565523 10.1056/NEJMra001529

[B2] Ambrosetti M. Abreu A. Corrà U. Davos C. H. Hansen D. Frederix I. (2021). Secondary prevention through comprehensive cardiovascular rehabilitation: From knowledge to implementation. 2020 update. A position paper from the Secondary Prevention and Rehabilitation Section of the European Association of Preventive Cardiology. *Eur. J. Prev. Cardiol.* 28 460–495. 10.1177/2047487320913379 33611446

[B3] Arduini A. Gomez-Cabrera M.-C. Romagnoli M. (2011). Reliability of different models to assess heart rate recovery after submaximal bicycle exercise. *J. Sci. Med. Sport* 14 352–357. 10.1016/j.jsams.2011.02.012 21450521

[B4] Avila A. Claes J. Buys R. Azzawi M. Vanhees L. Cornelissen V. (2020). Home-based exercise with telemonitoring guidance in patients with coronary artery disease: does it improve long-term physical fitness? *Eur. J. Prev. Cardiol.* 27 367–377. 10.1177/2047487319892201 31787026

[B5] Balady G. J. Williams M. A. Ades P. A. Bittner V. Comoss P. Foody J. M. (2007). Core Components of Cardiac Rehabilitation/Secondary Prevention Programs: 2007 Update: A Scientific Statement From the American Heart Association Exercise, Cardiac Rehabilitation, and Prevention Committee, the Council on Clinical Cardiology; the Councils on Cardiovascular Nursing, Epidemiology and Prevention, and Nutrition, Physical Activity, and Metabolism; and the American Association of Cardiovascular and Pulmonary Rehabilitation. *Circulation* 115 2675–2682. 10.1161/CIRCULATIONAHA.106.180945 17513578

[B6] Batalik L. Dosbaba F. Hartman M. Batalikova K. Spinar J. (2020). Benefits and effectiveness of using a wrist heart rate monitor as a telerehabilitation device in cardiac patients: A randomized controlled trial. *Medicine* 99:e19556. 10.1097/MD.0000000000019556 32176113 PMC7440288

[B7] Batalik L. Konecny V. Dosbaba F. Vlazna D. Brat K. (2021). Cardiac Rehabilitation Based on the Walking Test and Telerehabilitation Improved Cardiorespiratory Fitness in People Diagnosed with Coronary Heart Disease during the COVID-19 Pandemic. *IJERPH* 18:2241. 10.3390/ijerph18052241 33668304 PMC7956401

[B8] Cavalheiro A. H. Silva Cardoso J. Rocha A. Moreira E. Azevedo L. F. (2021). Effectiveness of Tele-rehabilitation Programs in Heart Failure: A Systematic Review and Meta-analysis. *Health Serv. Insights* 14 11786329211021668. 10.1177/11786329211021668 34188484 PMC8212368

[B9] Chan C. Yamabayashi C. Syed N. Kirkham A. Camp P. G. (2016). Exercise Telemonitoring and Telerehabilitation Compared with Traditional Cardiac and Pulmonary Rehabilitation: A Systematic Review and Meta-Analysis. *Physiother. Can.* 68 242–251. 10.3138/ptc.2015-33 27909373 PMC5125458

[B10] Corbett S. J. Ftouh S. Lewis S. Lovibond K. (2021). Acute coronary syndromes: summary of updated NICE guidance. *BMJ* 372:m4760. 10.1136/bmj.m4760 33452009

[B11] Cucinotta D. Vanelli M. (2020). WHO Declares COVID-19 a Pandemic. *Acta Bio. Medica. Atenei Parmensis* 91 157–160. 10.23750/abm.v91i1.9397 32191675 PMC7569573

[B12] Frederix I. Hansen D. Coninx K. Vandervoort P. Van Craenenbroeck E. M. Vrints C. (2015). Telerehab III: a multi-center randomized, controlled trial investigating the long-term effectiveness of a comprehensive cardiac telerehabilitation program - Rationale and study design. *BMC Cardiovasc. Disord.* 15:29. 10.1186/s12872-015-0021-5 25948479 PMC4432995

[B13] Gooley L. Gallagher R. Kirkness A. Bruntsch C. Roach K. Fletcher A. (2021). Remote Delivery of Cardiac Rehabilitation can Achieve Equivalent Health-related Quality of Life Outcomes to In-person Methods in Patients With Coronary Heart Disease During COVID-19: A Multi-site Study. *Heart Lung Circ.* 30:S283. 10.1016/j.hlc.2021.06.420

[B14] Haute Autorité de Santé. (2020). *Réponses Rapides dans le Cadre du COVID-19 Téléconsultation et Télésoin.* Available online at: https://www.has-sante.fr/upload/docs/application/pdf/2020-04/reponses_rapides_dans_le_cadre_du_covid-19_-teleconsultation_et_telesoin.pdf (accessed November 18, 2020).

[B15] Iliou M.-C. Pavy B. Martinez J. Corone S. Meurin P. Tuppin P. (2015). Exercise training is safe after coronary stenting: A prospective multicentre study. *Eur. J. Prev. Cardiol.* 22 27–34. 10.1177/2047487313505819 24057686

[B16] Janssen V. Gucht V. D. Dusseldorp E. Maes S. (2013). Lifestyle modification programmes for patients with coronary heart disease: a systematic review and meta-analysis of randomized controlled trials. *Eur. J. Prev. Cardiol.* 20 620–640. 10.1177/2047487312462824 23022703

[B17] Jolly M. A. Brennan D. M. Cho L. (2011). Impact of Exercise on Heart Rate Recovery. *Circulation* 124 1520–1526. 10.1161/CIRCULATIONAHA.110.005009 21947293

[B18] Jouven X. Courbon D. (2005). Heart-Rate Profile during Exercise as a Predictor of Sudden Death. *N Engl J Med* 8 1951–8 10.1056/NEJMoa043012 15888695

[B19] Karapolat H. Demir E. Bozkaya Y. T. Eyigor S. Nalbantgil S. Durmaz B. (2009). Comparison of hospital-based versus home-based exercise training in patients with heart failure: effects on functional capacity, quality of life, psychological symptoms, and hemodynamic parameters. *Clin. Res. Cardiol.* 98 635–642. 10.1007/s00392-009-0049-6 19641843

[B20] Kikuchi A. Taniguchi T. Nakamoto K. Sera F. Ohtani T. Yamada T. (2021). Feasibility of home-based cardiac rehabilitation using an integrated telerehabilitation platform in elderly patients with heart failure: A pilot study. *J. Cardiol.* 78 66–71. 10.1016/j.jjcc.2021.01.010 33579602

[B21] Kraal J. J. Peek N. Van den Akker-Van Marle M. E. Kemps H. M. (2014). Effects of home-based training with telemonitoring guidance in low to moderate risk patients entering cardiac rehabilitation: short-term results of the FIT@Home study. *Eur. J. Prev. Cardiol.* 21 26–31. 10.1177/2047487314552606 25354951

[B22] Lipinski M. J. Vetrovec G. W. Froelicher V. F. (2004). Importance of the first two minutes of heart rate recovery after exercise treadmill testing in predicting mortality and the presence of coronary artery disease in men. *Am. J. Cardiol.* 93 445–449. 10.1016/j.amjcard.2003.10.039 14969619

[B23] McDonagh T. A. Metra M. Adamo M. Gardner R. S. Baumbach A. Böhm M. (2021). 2021 ESC Guidelines for the diagnosis and treatment of acute and chronic heart failure. *Eur. Heart J.* 42 3599–3726. 10.1093/eurheartj/ehab368 34447992

[B24] Ministère des Solidarités et de la Santé (2020). *Arrêté du 10 Juillet 2020 Prescrivant les Mesures Générales Nécessaires pour Faire face à L’épidémie de Covid-19 dans les Territoires Sortis de L’état D’urgence Sanitaire et dans Ceux où il a été Prorogé.* Available online at: https://www.legifrance.gouv.fr/eli/arrete/2020/7/10/SSAZ2018110A/jo/texte

[B25] Pavy B. Barbet R. Carré F. Champion C. Iliou M.-C. Jourdain P. (2013). Therapeutic education in coronary heart disease: Position paper from the Working Group of Exercise Rehabilitation and Sport (GERS) and the Therapeutic Education Commission of the French Society of Cardiology. *Arch. Cardiovasc. Dis.* 106 680–689. 10.1016/j.acvd.2013.10.002 24239052

[B26] Pavy B. Iliou M.-C. Vergès-Patois B. Brion R. Monpère C. Carré F. (2012). French Society of Cardiology guidelines for cardiac rehabilitation in adults. *Arch. Cardiovasc. Dis.* 105 309–328. 10.1016/j.acvd.2012.01.010 22709472

[B27] Peçanha T. Bartels R. Brito L. C. Paula-Ribeiro M. Oliveira R. S. Goldberger J. J. (2017). Methods of assessment of the post-exercise cardiac autonomic recovery: A methodological review. *Int. J. Cardiol.* 227 795–802. 10.1016/j.ijcard.2016.10.057 27836300

[B28] Pelliccia A. Sharma S. Gati S. Bäck M. Börjesson M. Caselli S. (2021). 2020 ESC Guidelines on sports cardiology and exercise in patients with cardiovascular disease. *Eur. Heart J.* 42 17–96. 10.1093/eurheartj/ehaa605 32860412

[B29] Pericàs J. M. Hernandez-Meneses M. Sheahan T. P. Quintana E. Ambrosioni J. Sandoval E. (2020). COVID-19: from epidemiology to treatment. *Eur. Heart J.* 41 2092–2112. 10.1093/eurheartj/ehaa462 32511724 PMC7279517

[B30] Piepoli M. F. Hoes A. W. Agewall S. Albus C. Brotons C. Catapano A. L. (2016). 2016 European Guidelines on cardiovascular disease prevention in clinical practice: The Sixth Joint Task Force of the European Society of Cardiology and Other Societies on Cardiovascular Disease Prevention in Clinical Practice (constituted by representatives of 10 societies and by invited experts)Developed with the special contribution of the European Association for Cardiovascular Prevention & Rehabilitation (EACPR). *Eur. Heart J.* 37 2315–2381. 10.1093/eurheartj/ehw106 27222591 PMC4986030

[B31] Price K. J. Gordon B. A. Bird S. R. Benson A. C. (2016). A review of guidelines for cardiac rehabilitation exercise programmes: Is there an international consensus? *Eur. J. Prev. Cardiol.* 23 1715–1733. 10.1177/2047487316657669 27353128

[B32] Roth G. A. Johnson C. Abajobir A. Abd-Allah F. Abera S. F. Abyu G. (2017). Global, Regional, and National Burden of Cardiovascular Diseases for 10 Causes, 1990 to 2015. *J. Am. Coll. Cardiol.* 70 1–25. 10.1016/j.jacc.2017.04.052 28527533 PMC5491406

[B33] Scherrenberg M. Wilhelm M. Hansen D. Völler H. Cornelissen V. Frederix I. (2021). The future is now: a call for action for cardiac telerehabilitation in the COVID-19 pandemic from the secondary prevention and rehabilitation section of the European Association of Preventive Cardiology. *Eur. J. Prev. Cardiol.* 28 524–540. 10.1177/2047487320939671 PMC7928994 32615796

[B34] Scrutinio D. Temporelli P. L. Passantino A. Giannuzzi P. (2009). Long-term secondary prevention programs after cardiac rehabilitation for the reduction of future cardiovascular events: focus on regular physical activity. *Future Cardiol.* 5 297–314. 10.2217/fca.09.12 19450055

[B35] Shetler K. Marcus R. Froelicher V. F. Vora S. Kalisetti D. Prakash M. (2001). Heart rate recovery: validation and methodologic issues. *J. Am. Coll. Cardiol.* 38 1980–1987. 10.1016/S0735-1097(01)01652-7 11738304

[B36] Silva-Cardoso J. González Juanatey J. R. Comin-Colet J. Sousa J. M. Cavalheiro A. Moreira E. (2021). The Future of Telemedicine in the Management of Heart Failure Patients. *Card Fail Rev.* 7:e11. 10.15420/cfr.2020.32 34136277 PMC8201465

[B37] Taylor R. S. Dalal H. Jolly K. Zawada A. Dean S. G. Cowie A. (2015). “Home-based versus centre-based cardiac rehabilitation,” in *Cochrane Database of Systematic Reviews*, (Chichester, UK: John Wiley & Sons, Ltd), The Cochrane Collaboration. 10.1002/14651858.CD007130.pub3 26282071

[B38] Taylor R. S. Dalal H. M. McDonagh S. T. J. (2021). The role of cardiac rehabilitation in improving cardiovascular outcomes. *Nat Rev Cardiol* 16 1–15. 10.1038/s41569-021-00611-7 34531576 PMC8445013

[B39] Vysoký R. Fiala J. Dosbaba F. Bat’alík L. Nehyba S. Ludka O. (2015). Preventive Training Programme for Patients after Acute Coronary Event - Correlation between Selected Parameters and Age Groups. *Cent. Eur. J. Public Health* 23 208–213. 10.21101/cejph.a4125 26615651

[B40] Wisløff U. Støylen A. Loennechen J. P. Bruvold M. Rognmo Ø Haram P. M. (2007). Superior Cardiovascular Effect of Aerobic Interval Training Versus Moderate Continuous Training in Heart Failure Patients: A Randomized Study. *Circulation* 115 3086–3094. 10.1161/CIRCULATIONAHA.106.675041 17548726

[B41] Writing Committee, Eacpr, Guazzi M. Adams V. Conraads V. Halle M. (2012). Clinical recommendations for cardiopulmonary exercise testing data assessment in specific patient populations. *Eur. Heart J.* 33 2917–2927. 10.1093/eurheartj/ehs221 22952138

[B42] Zhang Y. Cao H. Jiang P. Tang H. (2018). Cardiac rehabilitation in acute myocardial infarction patients after percutaneous coronary intervention: A community-based study. *Medicine* 97:e9785. 10.1097/MD.0000000000009785 29465559 PMC5841979

